# Behavioral and Neuronal Characterizations, across Ages, of the TgSwDI Mouse Model of Alzheimer’s Disease

**DOI:** 10.3390/genes15010047

**Published:** 2023-12-28

**Authors:** Natalie A. Tan, Angelica M. Alvarado Carpio, H. Craig Heller, Elsa C. Pittaras

**Affiliations:** Department of Biology, Stanford University, Stanford, CA 94305, USA; ntan23@stanford.edu (N.A.T.); avocado@stanford.edu (A.M.A.C.); hcheller@stanford.edu (H.C.H.)

**Keywords:** Alzheimer’s disease, TgSwDI mouse model, β-amyloid, immunochemistry, memory, motor skills, aging, neurodegenerative diseases, longitudinal study

## Abstract

Alzheimer’s disease (AD) is a neurodegenerative disorder that currently affects as many as 50 million people worldwide. It is neurochemically characterized by an aggregation of β-amyloid plaques and tau neurofibrillary tangles that result in neuronal dysfunction, cognitive decline, and a progressive loss of brain function. TgSwDI is a well-studied transgenic mouse model of AD, but no longitudinal studies have been performed to characterize cognitive deficits or β-amyloid plaque accumulation for use as a baseline reference in future research. Thus, we use behavioral tests (T-Maze, Novel Object Recognition (NOR), Novel Object Location (NOL)) to study long-term and working memory, and immunostaining to study β-amyloid plaque deposits, as well as brain size, in hippocampal, cerebellum, and cortical slices in TgSwDI and wild-type (WT) mice at 3, 5, 8, and 12 months old. The behavioral results show that TgSwDI mice exhibit deficits in their long-term spatial memory starting at 8 months old and in long-term recognition memory at all ages, but no deficits in their working memory. Immunohistochemistry showed an exponential increase in β-amyloid plaque in the hippocampus and cortex of TgSwDI mice over time, whereas there was no significant accumulation of plaque in WT mice at any age. Staining showed a smaller hippocampus and cerebellum starting at 8 months old for the TgSwDI compared to WT mice. Our data show how TgSwDI mice differ from WT mice in their baseline levels of cognitive function and β-amyloid plaque load throughout their lives.

## 1. Introduction

Alzheimer’s disease (AD) is a progressive and irreversible neurodegenerative brain disease for which no treatment exists yet. In 2020, there were as many as 50 million AD patients worldwide and this number is projected to double every 5 years and will increase to 152 million by 2050 [1]. Thus, AD has been deemed a world public health concern and a major research priority for our society [2]. Understanding the mechanisms of AD as well as finding new treatments are important goals.

AD is believed to occur when abnormal amounts of β-amyloid (Aβ) accumulate outside neurons as amyloid plaques, and neurofibrillary tangles consisting of tau proteins form inside the neurons. Aβ is generated from the cleavage of the amyloid precursor protein (APP) by β-secretase and then by the γ-secretase complex into Aβ1-40 and Aβ1-42 peptides [3]. These peptides ultimately form both soluble and insoluble Aβ plaques seen in AD. Due to their defective clearance, Aβ proteins affect neuronal functioning and connectivity, resulting in a progressive loss of brain function [4] and cell death [2]. These functional losses can greatly impair cognitive function, impacting memory, learning, knowledge, reasoning, and other cognitive processes [5].

Aβ plaques are highly diverse structures that misfold and, by self-assembly, are believed to propagate by a prion-like mechanism [6]. The combination of inflammation, environmental triggers, and genetic variations have been linked to plaque formation and AD, but no single factor has emerged as the cause of AD [6]. Uncertainty about the mechanism of AD requires more research into the pathology of the disease, particularly to elucidate what potential long-term therapies might be viable. Much of this research requires animal models.

TgSwDI (APP-Swedish, Dutch, Iowa) mice are a well-studied mouse model of AD with the genetic background of the wild-type mouse C57BL/6. They express the human *amyloid precursor protein* (*APP*) gene (isoform 770) containing the Swedish (K670N/M671L), Dutch (E693Q), and Iowa (D694N) mutations under the control of the mouse Thy1 promoter [7]. The Swedish *APP* mutation increases the production of Aβ40/42 peptides, and the Dutch and Iowa *APP* variants each produce the same phenotype that predominantly accumulates Aβ40 [8]. In contrast to other AD mouse models, Aβ aggregation occurs rapidly and abundantly in TgSwDI with a physiological expression of transgenic human APP that is approximately 50% of the expression of the endogenous mouse APP [8,9,10,11,12,13,14]. This suggests that this model may be especially useful for investigating Aβ aggregation mechanisms, degradation, clearance, and the role that Aβ aggregation plays in the pathology of AD. Some studies have found that TgSwDI mice develop Aβ deposition at 3 to 12 months old [9]. Other studies show that Aβ is detectable and begins to accumulate at 2–3 months of age, and Aβ deposits are extensive by 12 months of age [8,10,15,16]. At 12 and 15 months old, immunohistochemistry reveals extensive Aβ plaque numbers in the hippocampus and neocortex [17], and at 15 months in the thalamus [11]. In TgSwDI mice at 20 months old, ELISA showed a presence of both soluble and insoluble Aβ-42 and Aβ-40 in both the hippocampus and the neocortex [13]. At 12 months old, extensive Aβ plaque was observed, with mainly diffuse parenchymal plaques in the neocortex and microvascular plaques in the hippocampus and thalamus [14].

The sizes of specific parts of the brain are also known to be relatively smaller in the brains of people with AD due to cerebral atrophy, a common feature of neurodegenerative disorders [18]. In particular, hippocampal volume decreases more acutely compared to other areas of the brain in cases of dementia [19]. Thus, we also measured the sizes of the hippocampus and cerebellum in the TgSwDI model mice at different ages to compare to the control mice brain slices.

Studies of cognitive deficits in TgSwDI mice have used a battery of tests, including the Novel Object Recognition (NOR), T-Maze, Barnes Maze, Morris Water Maze, Radial Arm Maze, and Rotarod. TgSwDI mice show learning and memory deficits, starting at 3–4 months old, in the Barnes Maze task and Morris Water Maze, which test spatial learning and memory. No differences in mobility, strength, or coordination were reported [7,20]. Other studies also showed memory deficits from around 7 to 20 months old in the Radial Arm Maze, Novel Object Recognition, or T-Maze [13,21,22,23,24,25,26,27,28]. In other studies, the control and TgSwDI mice showed the same performances in the Barnes Maze task or NOR at 3 months old [20,29,30]. Therefore, the timing at which these deficits appear still needs to be studied and determined. In the Rotarod test, TgSwDI showed deficits in performance compared to the control mice when tested from 7 months old [26,27]. This suggests that they have some deficits in their motor abilities, but more research into the specific ages must be performed to chart these deficits over time.

Current research into the behavior, cognition, and development of Aβ plaques of the TgSwDI mice is both contradictory at times and incomplete in terms of age. There are many previous studies on the TgSwDI model’s cognitive deficits, but the results taken together do not characterize the deficits in the context of aging, even though AD is a neurodegenerative disease with symptoms that progress over time. Furthermore, studies have shown that the timing of treatment of AD seems to be crucial, as the late treatment of AD shows little efficacy [31]. To develop efficacious treatments based on the understanding of the importance of timing, more studies need to be performed to minimize the window between the ages in which we have available data to effectively characterize this mouse model. To our knowledge, no longitudinal studies have been performed on the TgSwDI mice with the purpose of characterizing the evolution of the cognitive deficits alongside the accumulation of Aβ deposits in the neocortex, hippocampus, and thalamus for comparison. Therefore, we studied the spatial memory, recognition memory, working memory, mobility, and coordination of Tg-SwDI mice as well as Aβ deposits in their brains and the sizes of different areas of the brain at 3, 5, 8, and 12 months old.

## 2. Materials and Methods

### 2.1. Animals

Tg-SwDI mice were maintained at 23 ± 2 °C on a 12:12 light–dark schedule (light ON at 8:00 am) and had access to food and water ad libitum. All experimental procedures were approved by the Stanford University IACUC and were conducted in compliance with the NIH Guide for the Care and Use of Laboratory Animals. Efforts were made to minimize the number of animals used and to minimize their discomfort, as our aim was to study the behavior of non-stressed animals.

The Tg-SwDI mice were genotyped after breeding by using 2 µL of 10X advantage PCR buffer (Takara, San Jose, CA, USA, S1799), 0.4 µL of 10 mM dNTP Mix (Thermo Fisher Scientific, Burlington, ON, Canada, R0192), 0.14 µL of Primer Mix (20 µM: 10 µL of F primer: [AGGACTGACCACTCGACCAG], 10 µL of R primer [CGGGGGTCTAGTTCTGCAT] in 80 µL of water), and 0.2 µL of 50× Advantage 2 polymerase mix (from Takara/Clontech, Mountain View, CA, USA 639201) in 15.25 µL of water per 2 µL of DNA sample previously digested for a few hours in 192 µL of Direct PCR Mouse Tail Lysis Reagent (Viagen, San Jose, CA, USA, 102-T) with 8 µL of proteinase K solution (10 mg/mL). After running the thermocycler program (94 degrees for 3 min, 30 cycles of 94 degrees for 30 s, 62 degrees for 1 min, 72 degrees for 1 min, 72 degrees for 2 min, and 10 degrees for 1 min), the samples with 3 µL of 10× loading dye were run in 1.5% gel at 100–130 V. The control mice were the mice genotyped that did not show the presence of the human mutant *APP* gene.

Using the control and TgSwDI mice, we aimed to study the emergence of AD markers and cognitive deficits. Mice mature into adulthood at 3 months [32], and in order to study the initial development of the disease, we chose 12 months, which is the stage of middle to late adulthood [32], as the end of the study timeline.

Behavioral and immunohistochemistry experiments were conducted at 3, 5, 8, and 12 months of age. Different sets of mice of both genotypes went through the four behavioral tests at each of the age categories. The use of different mice each time was to prevent habituation to the tasks presented. The order of the behavioral tests was Novel Object Recognition, T-Maze, Novel Object Location, and Rotarod, which occurred over a period of 3 weeks. Furthermore, the mice that underwent behavioral tests were separated from the mice used in the immunohistochemistry experiments, since the timeline of behavioral experiments required almost a month of experimentation.

### 2.2. Behavioral Experiments

Mice of both genotypes went through the four behavioral tests only once at 1 of the ages: 3, 5, 8, or 12 months old. This was to prevent habituation to the tasks presented. 

#### 2.2.1. Working Memory: T-Maze

**Figure 1:**
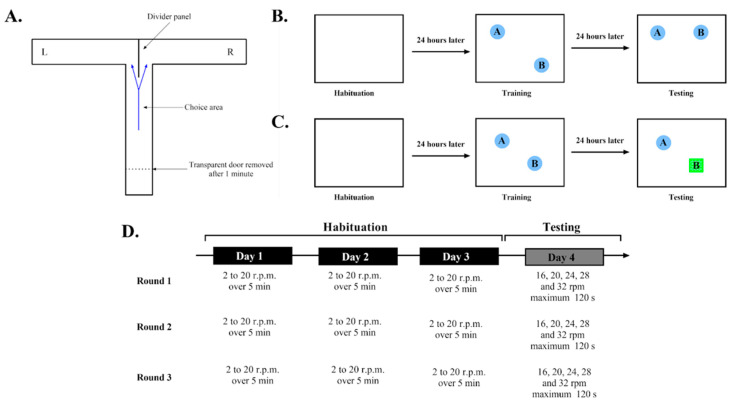
(**A**) Protocol of the T-maze. Schematic representation of the T-maze used during the working memory task. For one minute, the mouse is confined behind the transparent door and then permitted to explore the maze for 7 min. An alternation is counted when the animal visits the arm on the left (L) and then the arm on the right (R) or vice versa. (**B**) Protocol of the Novel Object Location (NOL). The NOL includes a 10 min habituation period, followed by a 10 min training period, during which the mouse can explore two identical objects, and a 7 min testing, during which one object is placed at a new location; each test was separated by 24 h. The blue circular objects A and B in the schematic represent two distinct, but equal objects. Object B is later moved to a different location. (**C**) Protocol of the Novel Object Recognition (NOR) test. The NOR includes a 10 min habituation period, followed by a 10 min training period, during which the mouse can explore two identical objects, and a 7 min test, during which one object is replaced by a new one; each test was separated by 24 h. The blue circular objects A and B represent two distinct, but equal objects. Blue circular object B is later replaced by green square object B, signifying a change in object shape and color. (**D**) Protocol of the Rotarod performance test. The Rotarod performance test lasted for four days: three days of habituation (from 2 to 20 r.p.m. over 5 min) and one day of testing (16, 20, 24, 28, and 32 r.p.m. for a maximum of 120 s).

The T-maze is based on the natural tendency of rodents to alternate between the left and right arms of a maze in the shape of a T [33,34,35]. This phenomenon is called spontaneous alternation. During this test, the mouse was first confined for 60 s in a start box at the beginning of the stem arm of a transparent plastic T-maze (48 cm × 7.1 cm × 20 cm). The start box (15 cm × 7.1 cm × 20 cm) was separated from the rest of the maze by a transparent door. After the door was opened, the animal was permitted to explore the maze for 7 min. A divider panel (15.4 cm × 20 cm, Figure 1A) was centered at the intersection of the ‘‘T’’ so that it extended 8.3 cm into the stem arm to force the animal to make a choice between the left and right arms. The maze was cleaned between each run with 10% ethanol.

The successive animal choices were recorded by hand at the same time as the test was being performed. An alternation attempt was scored when at least ⅓ of the body of the mouse entered 1 of the lateral arms, re–entered the stem arm, and then entered the lateral arm opposite the one previously chosen. Re-entry into the same arm was a non-alternation. The percentage of alternations was calculated as (number of alternations/total alternation attempts) × 100. Typically, we observed between 60 to 70% of the alternations and an animal that was non-alternating should have had a score close to 50% or less.

#### 2.2.2. Long-Term Spatial Memory: Novel Object Location (NOL)

The Novel Object Location (NOL) task is based on the tendency of rodents to prefer investigating new elements of their environment more than familiar ones [36,37]. Each test included a habituation phase, training phase, and testing phase preceded by one week of handling. During the 1st day (24 h before the training phase), the animals were individually familiarized for 10 min with an empty arena (white, walled, open field: 75 × 75 × 37 cm). During the training phase (2nd day), animals were given the opportunity to explore for 10 min 2 identical objects placed at the same distance from the walls and corners of the arena. For these experiments, the walls of the arenas had distinctive markings. We expected that time spent exploring each object would be approximately the same; otherwise, there was the possibility that the mouse displayed specific object preference or avoidance, and that mouse was eliminated from the study (over 65% or under 35% of their time close to any object during training). If the training session went well, the testing phase occurred 24 h later. During the testing phase, the mouse was placed in the same arena for 7 min, but 1 of the 2 objects was placed in a different location (Figure 1B). The arenas were cleaned with 10% ethanol between tests to ensure that no odor cues were carried over between sessions.

Real-time video recordings were obtained for the data acquisition during the training and testing sessions using the View-Point VideoTrack system (Montreal, QC, Canada). The data analysis included determinations of time spent close to each of the 2 objects during the training (10 min) and testing (7 min). We then calculated the discrimination indices representing the percentage of time spent around the object with a new location (object B) compared to the time spent around the other object placed in the same location as during the training session (object A, Figure 1B).

% DI = ((Time B)/(Time A + B)) * 100

#### 2.2.3. Long-Term Recognition Memory: Novel Object Recognition (NOR)

The Novel Object Recognition (NOR) experiments were performed exactly as the NOL task (Figure 1C), except:

1. During the testing phase, one of the two objects from the training phase was replaced by a novel object (object B) at the same location as one of the original objects explored during the training phase;
2. No object was moved to a different location.

The habituation phase and data acquisitions were performed in the same way as the NOL task. Regarding the data analysis, we measured the time the mouse spent around each object during the training (10 min) and testing (7 min) phases. We then calculated the discrimination index, which represented the percentage of time spent around the new object compared to the time spent around the familiar object (Figure 1C).

% DI = ((Time B)/(Time A + B)) * 100

As for the NOL, mice that demonstrated over 65% or under 35% preference scores for any object in the training session were excluded from the experiment.

#### 2.2.4. Motor Coordination: Rotarod Performance Test

The Rotarod performance test was used to study the motor skills of the mice [38,39,40]. It consisted of a 3 cm diameter rod that could rotate at different speeds.

The animals were pre-trained on an automated 4-lane Rotarod unit [41] that could be set to a fixed speed or accelerating speed. For the habituation phase, the mice were placed on a rod that accelerated smoothly from 2 to 20 r.p.m. over a period of 5 min. This habituation was performed three times a day for three days.

For the testing phase, the mice were placed on the rod and sequentially tested at 16, 20, 24, 28, and 32 rpm for a maximum of 120 s at each speed. The animals were tested 3 times at each speed with a rest period of 10 min between each trial (Figure 1D).

For both the habituation and testing phases, the length of time that each animal was able to stay on the rod was recorded as the latency to fall, registered automatically by a trip switch under the floor of each rotating drum. For the testing phase, we also recorded the r.p.m during which the mouse fell.

### 2.3. Cresyl Violet Staining and Immunohistochemistry

#### 2.3.1. Brain Removal and Conservation

The animals were anesthetized (isoflurane) and directly perfused transcardially with 20 mL of phosphate-buffered saline (PBS) and then by 50 mL of 4% paraformaldehyde (PFA). Brains were removed, fixed over 24 h with PFA, and then frozen at −20 °C.

#### 2.3.2. Brain Sections

Brains were sectioned with a cryostat (Cryostar NX70, ThermoFisher) on a coronal plane into 30 μm sections. We placed 1 out of every 5 sections on a slide for cresyl violet staining and 1 out of every 5 in PBS for immunohistochemistry.

#### 2.3.3. Immunohistochemistry (Figure 2)

Step 1. The brain sections were rinsed 3 times in PBS 1× for 5 min each time.
Step 2. To neutralize endogenous peroxidases, sections were placed in PBS 1X containing 3% H_2_O_2_ for 20 min.
Step 3. To block the non-specific site, we used a PBS 1X solution with 30% of donkey serum, 3% of H_2_O_2_, and 0.3% Triton (solution 1) for 30 min.
Step 4. Aβ immunohistochemistry was performed with a purified monoclonal mouse anti-Aβ antibody (anti-β amyloid 1-42 antibody [mOC64], BioLegend, #AB201060-1002) diluted at 1:100 in solution 1 for 24 h. Step 1 was then repeated.
Step 5. Sections were placed in solution 1 without Triton and a biotinylated secondary antibody (goat anti-rabbit IgG H&L (Biotin), BioLegend, #AB6720-1001) for 2 h. Step 1 was then repeated.
Step 6. Sections were then placed in solution 1 without Triton, 15 µL of solution A, and 15 µL of solution B from the ABC kit (Vectastain, standard, PK6100) for 30 min and screened from the light. Step 1 was then repeated.
Step 7. Sections were rinsed twice in Tris-HCl for 10 min each time.
Step 8. Sections were placed in a mixture of 1 tab of DAB (3, 3′-diaminobenzidine) and 45 mL of Tris-HCl for 5 min. Aβ samples were then revealed with 5µL per well of 3% of H_2_O_2_. Step 7 was then repeated. Step 1 was subsequently repeated.
Step 9. Sections were then placed on slides, allowed to dry, and then cover slipped with Cytoseal gel XYL (Epredia, ref 8312-4) and VWR microcover glass (VWR, ref 48404-454).

#### 2.3.4. Cresyl Violet Staining

The slides were immersed in different solutions for one minute, in the same order, before being covered with Cytoseal gel XYL (Epredia, Kalamazoo, MI, USA, ref 8312-4) and VWR microcover glass (VWR, Radnor, PA, USA ref 48404-454). The solutions in which the slides were emerged are as described below:

1. 95% ethanol.
2. 70% ethanol.
3. dH_2_O.
4. Cresyl violet stain.
5. dH_2_O.
6. 70% ethanol.
7. 95% ethanol.
8. 95% ethanol + 1% acetic acid.
9. 100% ethanol.
10. Xylene.

#### 2.3.5. Image Acquisition and Analysis

Brain sections used for immunohistochemistry, taken from between −1.94 and -2.46 mm from the bregma, were analyzed under an Olympus microscope (HB-2) at 40× magnification. Images were taken using the microscope in conjunction with QCapture Pro 7 and analyzed using ImageJ v. 1.53 software. The scale was added and the Aβ plaque area in each section was measured using the ImageJ freehand selection and “measure” function. The total area was kept consistent between slices such that the area taken into consideration between slices was the same.

The size of the brain was measured by manually delimiting the hippocampus and cerebellum when the coronal slices were close to −1.58 mm from the bregma and the sagittal slices were close to −0.04 mm in a lateral position. After the brain areas were delimited by using the freehand selection of ImageJ, we added the scale and measured the area by using the function “measure” in Image J. Images were taken using QCapture at 10× magnification.

### 2.4. Statistics

Statistical analyses were performed using Statview v. 5.0 software. If the data showed a normal distribution (Shapiro–Wilk test) and passed equal variance tests (*F* test), statistical analyses were performed using a *t*-test or analysis of variances (ANOVAs). If not, statistical analyses were performed using Wilcoxon task sign-rank, Kruskal–Wallis, or Mann–Whitney tests. For the behavioral tests and neurochemical results, ANOVAs were used to test the effects of genotype, time (training vs. testing), and the combined effect of genotype × time. *p*-values ≤ 0.05 were considered statistically significant.

## 3. Results

### 3.1. T-Maze

**Figure 3:**
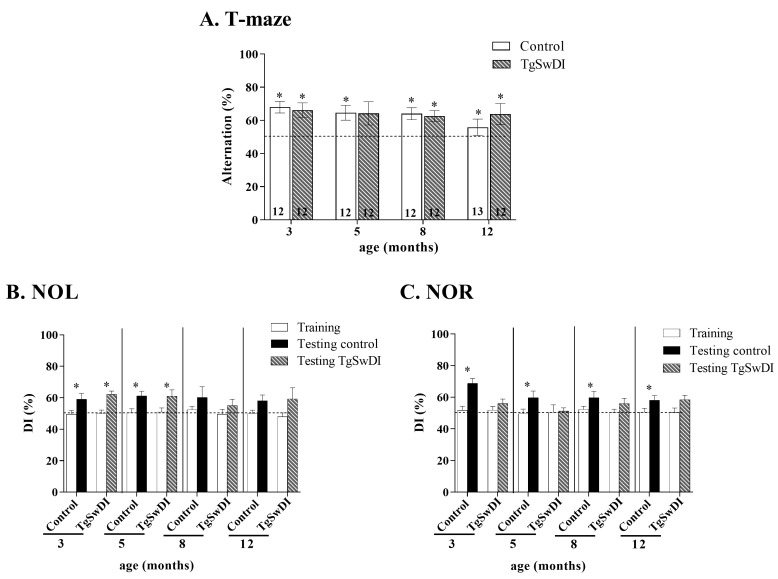
(**A**) T-maze results. Percentage of alternations during the T-maze test for the 3-, 5-, 8-, and 12-month-old control (solid bars) and Tg-SwDI (striped bars) mice. *, *p* < 0.05 compared to 50% of alternations represented by the dotted line. Number of mice by group is indicated inside the histogram. (**B**) NOL results. Percentage discrimination index (DI) values for the Novel Object Location (NOL) test for the 3-, 5-, 8-, and 12-month-old control (solid bars) and Tg-SwDI (striped bar) mice. All groups contain 12 mice, except the control mice at 12 years old that contained 13 mice. *, *p* < 0.05 comparison between training and testing. The chance level is represented by the dotted line. (**C**) NOR results. Percentage discrimination index (DI) values for the Novel Object Recognition (NOR) test for the 3-, 5-, 8-, and 12-month-old control (solid bars) and Tg-SwDI (striped bar) mice. All groups contain 12 mice. *, *p* < 0.05 comparison between training and testing. The chance level is represented by the dotted line.

A two-way ANOVA revealed no effects of genotype, age, no a combined effect of genotype and age. As shown in Figure 3A, all mice, except the 5-months-old TgSwDI mice, showed significant spontaneous alternation behaviors compared to the random chance alternation at 50% during the T-maze test. The 5-months-old TgSwDI mice only showed a tendency to alternate (control: 3 months: Z = −2.981, p = 0.002; 5 months: Z = −2.471, p = 0.01; 8 months: Z = −2.666, p = 0.007; 12 months: Z = −2.666, p = 0.007; TgSwDI: 3 months: Z = −2.667, p = 0.007; 5 months: Z = −1.804, p = 0.07; 8 months: Z = −2.266, p = 0.007; 12 months: Z = −2.000, p = 0.04).

### 3.2. NOL

A two-way ANOVA revealed no significant effects of genotype, age, or any combined effect of genotype and age. As shown in Figure 3B, only the 3- and 5-months-old mice in both genotypes exhibited long-term spatial memory during the Novel Object Location test, while the 8- and 12-month-old mice in both genotypes showed significant deficits in their long-term spatial memory.

(Control: 3 months: Z = −2.040, p = 0.04, 5 months: Z = −2.667, p = 0.007; TgSwDI: 3 months: Z = −2.746, p = 0.006; 5 months: Z = −2.197, p = 0.02).

### 3.3. NOR

A two-way ANOVA revealed a significant effect of genotype (F (1.63) = 11.354, p = 0.002) as well as a combined effect of age and genotype (F (1.3) = 2.665, p = 0.05). Only the control mice exhibited long-term recognition memory during the Novel Object Recognition test, while TgSwDI mice showed significant deficits in their long-term recognition memory (control: 3 months: Z = −2.824, p = 0.004; 5 months: Z = −2.341, p = 0.01; 8 months old: t = −2.317, p = 0.04; 12 months: Z = −2.401, p = 0.01; Figure 3C). 

### 3.4. Rotarod

**Figure 4:**
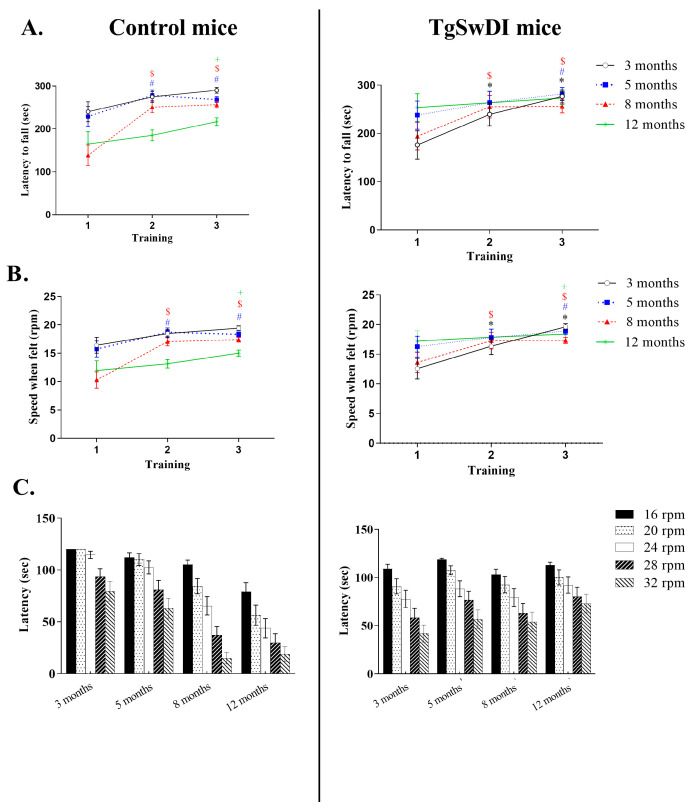
Rotarod results for training (**A**,**B**) and testing (**C**) of the 3-, 5-, 8-, and 12-month-old control and TgSwDI mice. All groups contained 12 mice, with the exception of the control mice at 12 years old, which contained 13 mice. *, *p* < 0.05 comparison between the control and TgSwDI mice. * represents significant difference from the first training for the 3 months old. # represents significant difference from the first training for the 5 months old. $ represents significant difference from the first training for the 8 months old. + represents significant difference from the first training for the 12 months old.

During the training stage, all mice improved regarding the latency to fall and the speed of rod at which they fell (Figure 4A,B). For the control mice, a two-way ANOVA revealed a significant effect of age, training, and a combined effect of training × age (latency to fall: F(3.88) = 5.132, p = 0.003; F(2.88) = 34.003, p < 0.0001; F(3.2) = 3.818, p = 0.002—speed at fall: F(3.88) = 5.119, p = 0.004; F(2.88) = 32.733, p < 0.0001; F(3.2) = 3.437, p = 0.004).

For the TgSwDI mice, a two-way ANOVA revealed a significant effect of training (latency to fall: F (2.90) = 19.886, p < 0.0001—speed at fall: F (2.90) = 18.013, p < 0.0001). In the same way, only an effect of age was observed for the control mice during the testing (F (3,176) = 10.156, p < 0.0001, Figure 4C).

By observing at the latency to fall during the testing, a significant effect of genotype was also revealed at 3 (F (1.92) = 6.893, p = 0.01) and 12 months old (F (1.88) = 9.084, p = 0.006, Figure 4C).

### 3.5. Immunohistochemistry Results

**Figure 5:**
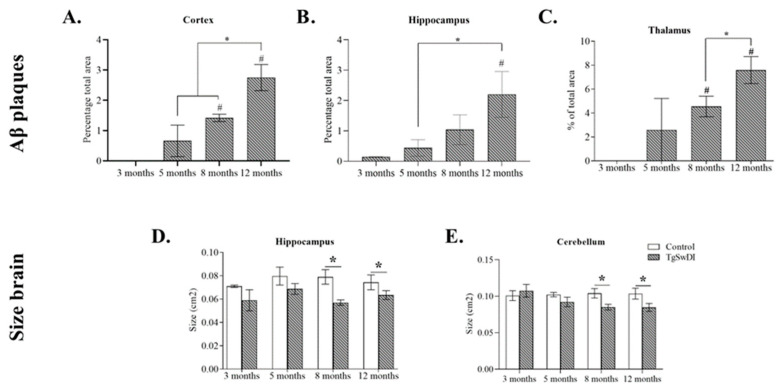
Results Aβ plaques. Percentages of total area covered by Aβ plaque in the cortex (**A**), hippocampus (**B**), and the thalamus (**C**) of TgSwDI mice. # represents a difference from 3 months old and * represents a difference between the different ages. Results: size of brains. Comparison of sizes of the hippocampus (**D**) and cerebellum (**E**) for TgSwDI and control mice at 3, 5, 8, and 12 months. *, *p* < 0.05 between control and TgSwDI mice.

Aβ plaque aggregation was observed in TgSwDI mice at all ages, with plaques becoming larger and more numerous as the age increased (Figure 5A–C). No significant Aβ plaque levels were observed at any age for the control mice.

In the neocortex of the TgSwDI mice, the percentage of the total area covered by Aβ plaques was significantly larger at 12 months old than at 3 months old (3 months old vs. 8 months old: t = −12.975, p = 0.0002; 3 months old vs. 12 months old: t = −5.447, p = 0.001) and this amount of Aβ plaques was significantly higher at 12 months old than at 5 or 8 months old (5 months old vs. 12 months old: t = −3.520, p = 0.01; 8 months old vs. 12 months old: t = −2.692, p = 0.03). In the hippocampus, the percentage of the total area covered by Aβ plaques was significantly higher at 12 months old in the TgSwDI mice than at 3 months old (3 months old vs. 12 months old: t = −2.236, p = 0.02), and this value was significantly higher at 12 months old than at 5 months old (5 months old vs. 12 months old: t = −2.087, p = 0.03). In the thalamus, the percentage of the total area covered by Aβ plaques was significantly higher at 8 and 12 months old in the TgSwDI mice than at 3 months old (3 months old vs. 8 months old: t = −6.755, p = 0.002; 3 months old vs. 12 months old: t = −5.625, p = 0.001), and this value was significantly higher at 12 months old than at 8 months old (5 months old vs. 12 months old: t = −2.429, p = 0.05).

Aβ plaque aggregations were also significantly greater in the thalamus compared to the neocortex and the hippocampus at 8 (cortex: t = −4.550, p = 0.01; hippocampus: t = −4.479, p = 0.01) and 12 (cortex: t = −4.468, p = 0.002; hippocampus: t = −4.448, p = 0.002) months old in the TgSwDI mice.

**Figure 6:**
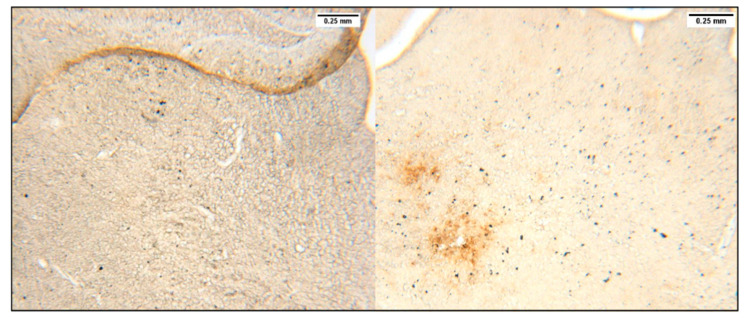
Immunohistochemistry. Thalamus of 12-month-old control mouse (**left**) and thalamus of 12-month-old TgSwDI mouse (**right**). A total of 14 TgSwDI (3 months old, *n* = 3; 5 months old, *n* = 3; 8 months old, *n* = 3; 12 months old, *n* = 5) and 17 control (3 months old, *n* = 3; 5 months old, *n* = 3; 8 months old, *n* = 4; 12 months old, *n* = 7) mice were used for immunochemistry.

Representative images are shown in Figure 6.

### 3.6. Hippocampus and Cerebellum Size Differences between Control and TgSwDI Mice

As shown in Figure 5D,E the sizes of the hippocampus and cerebellum of the TgSwDI mice did not differ from the control mice at 3 and 5 months old. At 8 and 12 months old, the sizes of the hippocampus and cerebellum were significantly smaller than the control mice (Hippocampus—8 months old: t = −3.708, p = 0.006; 12 months old: t = −2.572, p = 0.02. Cerebellum—8 months old: t = −3 = 2.804, p = 0.02; 12 months old: t = −2.272, p = 0.04). Repeated ANOVA measures revealed an effect of genotype only for the hippocampus (F (1.9) = 92.256, p = 0.002).

**Figure 7:**
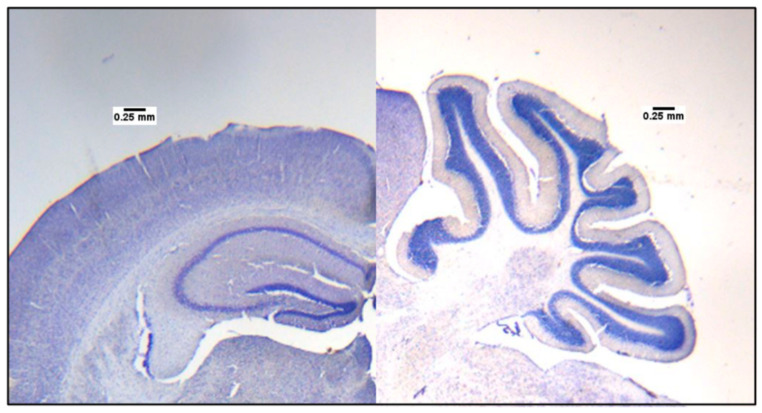
Sizes of cerebellum and hippocampus. A total of 24 TgSwDI (3 months old, *n* = 4; 5 months old, *n* = 8; 8 months old, *n* = 5; 12 months old, *n* = 7) and 21 control (3 months old, *n* = 4; 5 months old, *n* = 6; 8 months old, *n* = 5; 12 months old, *n* = 6) mice were used for cresyl violet staining. Examples of the hippocampus and cerebellum of a 12-month-old control mouse.

Representative images are shown in Figure 7.

## 4. Discussion

The present study characterized the longitudinal development of AD pathology in a TgSwDI mouse model of AD. We did this by investigating the cognitive deficits at each age (3, 5, 8, and 12 months old) through a series of behavioral tests. We further juxtaposed the cognitive deficits at each of these ages with the neuronal development of characteristic Aβ plaque areas in the hippocampus, neocortex, and thalamus of the TgSwDI mice.

No deficits in short-term working memory observed in TgSwDI at all ages.

No significant differences were observed between the TgSwDI and control mice in performance on the T-Maze test, suggesting that TgSwDI mice did not experience a deficit in their short-term working memory at any age. This is in conflict with the previous literature that suggests TgSwDI mice exhibit deficits in spontaneous alternation tests using the T-Maze [23]. However, this previous research was performed with a different protocol, where the mouse started from the base of the T-Maze, chose an arm, and was then gated in that arm for 30 s before being removed from the maze and returned to the base of the T-Maze manually [23]. We hypothesized that the difference in the protocols here could explain the differences between the data. Our mice showed no significant differences in spontaneous alternation, which could have occurred due to their ability to explore the maze uninterrupted for 7 min after 1 min of habituation. We handled our mice regularly, and this uninterrupted exploration allowed the mice to display their decision-making skills and cognitive abilities without any confounding factors due to human interference [42].

Deficits in long-term spatial memory occur later in life in TgSwDI mice, starting at 8 months old.

The behavioral data show that the TgSwDI mouse model of AD experiences varying levels of deficits as the mice age, with deficits in their long-term spatial memory at 8 months old and above. However, the control mice also exhibited deficits in their long-term spatial memory at 8 months old and above. Both genotypes were unable to discriminate significantly between objects at the old location and objects at a novel location at 8 and 12 months old (Figure 3A). The potential reasons for the control mice not discriminating between locations at 8 and 12 months old were two-fold. One of the control mice displayed a discrimination index of 20%, which skewed the average discrimination index of the control mice, eliminating significances in the control mice data at 8 and 12 months old *(t =* 2.344, *p* = 0.04). Another factor was the consideration that moving an object to another location may not have been as interesting and novel to the mice, and thus caused no significant differences in the exploration of objects at old vs. new locations. Other studies of spatial learning and memory using the Barnes Maze Task and Radial Arm Maze showed no spatial memory deficits at 3 months old, but long-term spatial memory deficits at 8 and 15 months old were evident [20,21,22,29]. We confirm these results with our NOL data that show no deficits at 3 and 5 months old, but deficits at 8 and 12 months old. Our data, in the context of the literature, suggest that long-term spatial learning deficits do not emerge until around 8 months of age and persist throughout the rest of the mouse’s lifespan.

Deficits in long-term recognition memory at all ages in TgSwDI mice, starting at 3 months old.

A significant difference was observed between the TgSwDI mouse model and control mice in the Novel Object Recognition test discrimination index at all ages. The TgSwDI mice did not discriminate significantly between the novel and familiar objects at any age, whereas the control mice discriminated significantly between objects at all ages tested (Figure 3C). This suggests that TgSwDI mice have deficits in their long-term recognition memory starting at 3 months old, which extends to the rest of their lifetime. This was consistent with the findings of previous studies that used NOR to test mice at 9, 14, and 18 months, and showed that TgSwDI mice did not explore the novel object at a significantly different level at any of those ages [24,25]. However, our findings contradict those from another study that shows that TgSwDI mice do discriminate between objects at 3–4 months [30]. We hypothesized that the deficit in the long-term recognition memory could start at that age range, which could explain studies achieving mixed results in that time frame.

TgSwDI mice experience latency in their motor development behavior at 3 months old.

Our Rotarod data show there is a significant difference in latency between TgSwDI and control mice at 3 months old, with TgSwDI mice demonstrating a deficit with a smaller latency to fall, but not at 5 or 8 months old (Figure 3D). Furthermore, there is a significant difference between genotypes at 12 months old as well, but with the control mice having a lower score (smaller latency to fall) compared with TgSwDI mice. As AD has been shown to affect motor function in humans over time [43], the TgSwDI mouse model may not display the exact same patterns of motor ability deficit development. However, the latency in motor development demonstrated in the TgSwDI mice in our Rotarod data is consistent with the emergence of motor function deficits before the onset of other AD symptoms in humans [43]. The present study showed that TgSwDI mice exhibited motor function deficits before the onset of spatial cognitive deficits and at the same time as recognition cognitive deficits.

TgSwDI mice develop Aβ plaques exponentially over time in the neocortex, hippocampus, and thalamus, starting at 3 and 5 months old.

Immunohistochemistry and subsequent imaging on cortical, hippocampal, and thalamic brain sections highlighted an exponential increase in Aβ plaque levels as the TgSwDI mice aged from 3 to 12 months old (Figure 5). Control mice exhibited almost no Aβ plaque levels at all ages and no patterns of increasing Aβ plaque formation either. Other studies confirm the presence of Aβ plaques at specific ages. At 12 and 15 months old, immunohistochemistry revealed extensive Aβ plaques in the hippocampus and neocortex [17], and at 15 months old in the thalamus [11]. At 13 months old, Aβ was isolated from the brain capillaries in higher concentrations compared to the WT mice [12]. In TgSwDI mice at 20 months old, ELISA showed the presence of both soluble and insoluble Aβ-42 and Aβ-40 plaques in both the hippocampus and neocortex [13]. At 12 months old, extensive Aβ plaques were observed, with mainly diffuse parenchymal plaques in the neocortex and microvascular plaques in the hippocampus and thalamus [14]. We therefore confirmed these results as well as narrowed the windows when and where those plaques appeared in TgSwDI mice.

Moreover, we observed significantly elevated thalamus Aβ plaque levels as compared to the neocortex and hippocampus Aβ plaque levels at 8 and 12 months old. This suggests that the thalamus may be an important area of study for Aβ plaque aggregation, AD pathogenesis and pathology, and Aβ clearance and aggregation mechanisms. The Aβ plaque levels in the hippocampus and neocortex slices were comparatively similar but impacted discrete types of cognitive deficits—these are discussed below.

Furthermore, it is hypothesized that Aβ spreads and aggregates by self-assembly through a prion-like mechanism, in which misfolded proteins propagate by inducing properly folded proteins into misfolding [6]. We observed in our immunohistochemistry experiments that Aβ plaque surface areas increased exponentially in the neocortex, hippocampus, and thalamus in TgSwDI mice. Exponential patterns of growth are a hallmark feature of prions in most environments [44]. This exponential pattern supports the hypothesis of the prion-like aggregation and formation of Aβ plaque. If this prion-like mechanism is indeed true, the elevated levels of Aβ plaques in the cortices as compared to the hippocampus may also point to a mechanism and direction of Aβ plaque aggregation across areas of the brain in TgSwDI mice. However, more research is needed to understand and test these hypotheses.

Cognitive deficits correlate with the location and level of Aβ plaque formation, and plaque formation precludes long-term spatial memory deficits in TgSwDI mice.

Previous research has shown that spatial memory is associated with hippocampal function, whereas recognition memory is associated with both the hippocampus and perirhinal neocortex function along with larger areas of the neocortex in general [45,46]. Our data show that TgSwDI mice exhibit significant deficits in their long-term recognition memory starting from 3 months old and deficits in their long-term spatial memory starting from 8 months old, after the levels of Aβ plaque have significantly increased. Given this outcome, it stands to reason that recognition memory, which relies on two areas of the brain that both develop Aβ plaques exponentially over time in TgSwDI mice, is affected significantly at all ages, and spatial memory is affected in a different, less severe, manner. Given that Aβ plaques are located in these two areas of particular interest, this suggests that cognitive deficits may be related to the location of Aβ plaque formation and deposition.

The lack of efficacy of potential treatments for AD in clinical trials is hypothesized to be due to relatively late treatment. This is thought to be because Aβ plaque development, Aβ deposits, and tau tangles, alongside damage to the brain, are shown to start a decade or more before cognitive decline in humans [31]. Therefore, the timing of Aβ plaque development as it relates to the timing of cognitive deficit development is important to understand the accuracy of this model for human AD. The current study shows that Aβ plaque deposits are seen at least 5 months before the onset of spatial memory deficits, which is consistent with the Aβ plaque deposits precluding cognitive decline in humans. Furthermore, we can see that recognition memory deficits occur as early as 3 months, the same time at which the formation of Aβ plaque deposits is also nascent.

Hippocampus and cerebellum sized in TgSwDI mice significantly decreased at 8 and 12 months old.

It is understood that the human brain changes and atrophies as it ages [18,47,48], and that the number of dopaminergic receptors decreases [47]. In AD, brain atrophy is accelerated, with specific areas affected at a greater rate [18]. The evidence shows that volume reductions in the hippocampus are different in patterns of healthy aging and in dementia, with longitudinal hippocampal maps showing deficits and regional tissue loss in comparison to healthy controls [19]. Our imaging from the cresyl violet staining protocol showed that TgSwDI mice exhibited significantly smaller hippocampus and cerebellum sizes in comparison with the control mice at 8 and 12 months old. These data are consistent with the literature on brain atrophy over time in human AD subjects and suggest that TgSwDI mice have similar brain atrophy patterns over time as seen for human AD.

While we know that Aβ plaque levels increase exponentially in mice aged 3–12 months old, the level of hippocampal atrophy seen in the TgSwDI mice at 12 months old can be lower than that seen in some AD patients at the analogous human age of ~60 years old (Figure 5D). We also observed that the decline in cognitive function was less severe than that seen for AD and did not increase with age at 8 and 12 months in the TgSwDI mouse model (Figure 3). However, 12 months represents middle to late adulthood in mice, so more hippocampal atrophy and further increasing cognitive decline may be seen even later in life. In the present study, we aimed at studying the emergence of AD markers and cognitive deficits. Therefore, we observed the progressive emergence of the disease, which explained why the progression of disease was less severe in TgSwDI mice at 12 months than in human AD patients. Ascertaining the late-stage effects of the disease in TgSwDI mice requires further experimentations at the 15-, 18-, 20-, and 24-month contexts.

**Figure 8:**
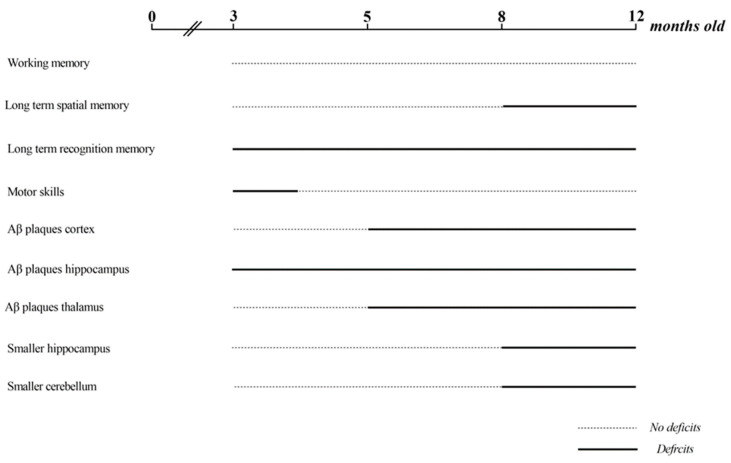
Summary. The dotted lines show when the TgSwDI mice did not show deficits, Aβ plaques, or smaller brain sizes. The solid lines show when TgSwDI mice showed deficits, Aβ plaques, or smaller brain sizes. At 3 months old, TgSwDI mice showed long-term recognition and motor skill deficits and started to show Aβ plaques in the hippocampus. At 5 months old, TgSwDI mice started to show Aβ plaques in the cortex and hippocampus. At 8 months old, TgSwDI mice started to show long-term spatial memory deficits as well as smaller hippocampuses and cerebellums than the control mice.

Taken together, our findings within the context of the literature suggest these conclusions: (1) TgSwDI mice exhibit cognitive deficits in their long-term recognition memory at all ages, no deficits in short-term working memory, and only partially in long-term spatial memory starting at 8 months; (2) TgSwDI mice develop Aβ plaques exponentially, as they age from 3–12 months old, in the neocortex, hippocampus, and thalamus; (3) cognitive deficits seen in TgSwDI mice may correlate with different areas of Aβ plaque formation in the brain, and plaque formation precludes some cognitive deficits in TgSwDI mice; and (4) TgSwDI mice have a smaller hippocampus and cerebellum, starting at 8 months old, than the control mice (Figure 5D,E). A summary timeline of these findings can be found in Figure 8. Thus, as a model of AD, the TgSwDI model has both strengths and weaknesses, and may be particularly useful in studying Aβ plaque aggregation mechanisms and the connection to AD pathology and pathogenesis, as well as long-term recognition memory deficit formation, progression, and treatment in AD. The study of the size of the brain with this mouse model is also particularly useful. However, it may not be the best model to study motor function or short-term working memory loss in AD due to the inconsistencies between the age at which motor deficits develop in mice vs. humans. In the context of these myriad topics, we also recommend that TgSwDI should further be used to study the function of the thalamus in AD pathogenesis and pathology, the function of Aβ in the proposed mechanisms of AD, and the hypothesized Aβ prion-like aggregation mechanism.

## Figures and Tables

**Figure 2:**
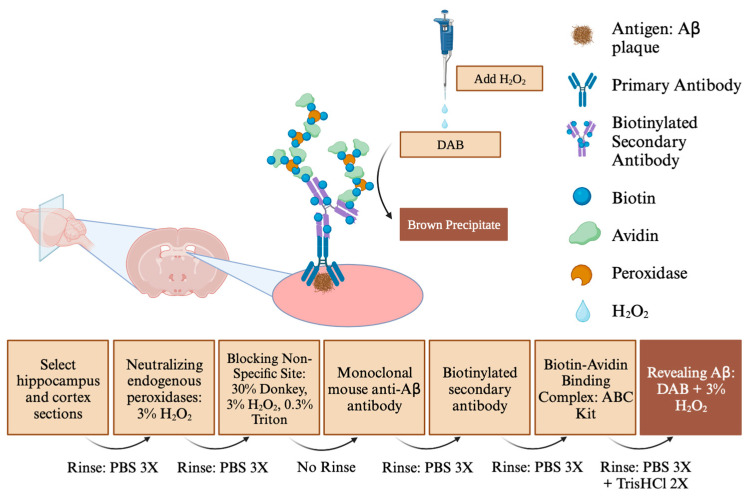
Protocol of biotin–avidin immunohistochemistry. Image created using BioRender: publication license available.

## Data Availability

The authors confirm that the data supporting the findings of this study are available within the article.
